# Discovery of global genomic re-organization based on comparison of two newly sequenced rice mitochondrial genomes with cytoplasmic male sterility-related genes

**DOI:** 10.1186/1471-2164-11-209

**Published:** 2010-03-29

**Authors:** Sota Fujii, Tomohiko Kazama, Mari Yamada, Kinya Toriyama

**Affiliations:** 1 Laboratory of Environmental Biotechnology, Graduate School of Agricultural Science, Tohoku University, 1-1 Tsutumidori-Amamiyamachi, Aoba-ku, Sendai 981-8555, Japan; 2 Current address: ARC Centre of Excellence in Computer Systems Biology, University of Western Australia, 35 Stirling Highway, Crawley 6009, WA, Australia

## Abstract

**Background:**

Plant mitochondrial genomes are known for their complexity, and there is abundant evidence demonstrating that this organelle is important for plant sexual reproduction. Cytoplasmic male sterility (CMS) is a phenomenon caused by incompatibility between the nucleus and mitochondria that has been discovered in various plant species. As the exact sequence of steps leading to CMS has not yet been revealed, efforts should be made to elucidate the factors underlying the mechanism of this important trait for crop breeding.

**Results:**

Two CMS mitochondrial genomes, LD-CMS, derived from *Oryza sativa *L. ssp. *indica *(434,735 bp), and CW-CMS, derived from *Oryza rufipogon *Griff. (559,045 bp), were newly sequenced in this study. Compared to the previously sequenced Nipponbare (*Oryza sativa *L. ssp. *japonica*) mitochondrial genome, the presence of 54 out of 56 protein-encoding genes (including pseudo-genes), 22 tRNA genes (including pseudo-tRNAs), and three rRNA genes was conserved. Two other genes were not present in the CW-CMS mitochondrial genome, and one of them was present as part of the newly identified chimeric ORF, CW-*orf307*. At least 12 genomic recombination events were predicted between the LD-CMS mitochondrial genome and Nipponbare, and 15 between the CW-CMS genome and Nipponbare, and novel genetic structures were formed by these genomic rearrangements in the two CMS lines. At least one of the genomic rearrangements was completely unique to each CMS line and not present in 69 rice cultivars or 9 accessions of *O. rufipogon*.

**Conclusion:**

Our results demonstrate novel mitochondrial genomic rearrangements that are unique in CMS cytoplasm, and one of the genes that is unique in the CW mitochondrial genome, CW-*orf307*, appeared to be the candidate most likely responsible for the CW-CMS event. Genomic rearrangements were dynamic in the CMS lines in comparison with those of rice cultivars, suggesting that 'death' and possible 'birth' processes of the CMS genes occurred during the breeding history of rice.

## Background

In contrast to the compact structure of the human mitochondrial genome (13 genes in approximately 17 kb), higher plant mitochondria are extremely complex in their constitution, and their process of evolution is enigmatic. In addition, although animal mitochondrial genomes are relatively conserved in size, from *Homo sapiens *to *Mus musculus*, the genomic content of plant mitochondria is highly divergent, ranging from an estimated 200 to 2,400 kb [1]. The conservation of simple circular mitochondrial DNA that is seen in animals is even in doubt in plants, and a number of studies have predicted that the genetic information of plant mitochondria is partitioned into subgenomes [2]. Finally, in addition to the 13 housekeeping genes also found in animals, plants contain numerous open reading flames (ORFs) with unknown functions as well as genes with known functions, such as ribosomal protein subunit-encoding genes, that are not observed in the animal genomes. Although most of these ORFs are considered to be non-functional and suppressed during development, some studies have reported that several ORFs are transcribed [3]. These mysteries of the mitochondria are certainly intriguing in view of the co-evolution of nuclear and mitochondrial genomes. To date, seven mitochondrial genomes have been sequenced from plant species: four from dicot species, *Arabidopsis thaliana *[4], *Beta vulgaris *[3,5], *Brassica napus *[6], and *Nicotiana tabacum *[7], and three from monocot species, *Oryza sativa *[8,9], *Zea mays *[10,11], and *Triticum aestivum *[12]. These studies have shown that plant mitochondrial genomes are actually divergent in their non-coding sequences and even in their gene-coding regions; for instance, the presence of ribosomal protein subunit-encoding genes is known to be quite inconsistent among species [2,13].

Cytoplasmic male sterility (CMS) is a maternally inherited male sterile trait, and a significant amount of evidence indicates that this phenomenon stems from alteration of the mitochondrial genome [14,15]. In many cases, the product of the unique gene structure in the CMS mitochondrial genome, consisting of a chimeric structure of endogenous gene fragments and an alien sequence, has been demonstrated to be associated with the mitochondrial inner membrane [16-19]. Most of these mitochondrial CMS-associated genes (MCAG) are thought to form a pore within the membrane and, although the details of the mechanism from pore-forming to CMS occurrence have not been fully elucidated, it is speculated that MCAGs are responsible for CMS. Since CMS is often associated with cytoplasmic substitutions, such as successive backcrossing of distantly related strains, CMS is the key phenomenon that may shed some light on plant mitochondrial-nuclear interactions.

Studies of the CMS mitochondrial genomes in *B. vulgaris *[3] and *Z. mays *[11] revealed the presence of several large recombination events compared to normal cytoplasm. Satoh et al. [3] sequenced the Owen-CMS mitochondrial genome in sugar beet, and a comparison with the non-CMS mitochondria revealed the existence of several CMS-specific ORFs generated by the genomic rearrangements. *PreSatp6 *was later determined to be the best candidate for MCAG, and the gene products of *PreSatp6 *were localized in the mitochondrial inner membrane in the form of a homo-oligomer [18]. Allen et al. [11] sequenced five distinct mitochondrial genomes in maize and detected massive genomic recombinations among the lines. Even two of the normal cytoplasms which were sequenced, NA and NB, were significantly dissimilar to each other, and it was estimated that there were 16 genomic rearrangements between the lines. They reported that genome sizes of the lines ranged from 535,825 bp in CMS-T to 739,719 bp in CMS-C [11].

The 490,520-bp mitochondrial genome of rice (*Oryza sativa *L.) *japonica *cultivar Nipponbare was sequenced, and 53 protein-encoding genes, 17 tRNA genes, and 3 rRNA genes were predicted [8]. When the existence of the master circle was assumed, at least three large duplications (>10,000 bp) were found, and portions of plastid and nuclear sequences were frequently inserted, making the genome even more complex. The mitochondrial genome of the *indica *strain 93-11 was also assembled, and it was predicted to possess 491,515-bp nucleotides [9]. At the same time, the 490,673-bp mitochondrial genome was assembled for the strain PA64S carrying *japonica *cytoplasm. A total of 96 SNPs and 25 Indels were predicted between 93-11 and PA64S, but the differences in their overall genomic structures were rather conservative, in comparison to the report that the sequence arrangements of two normal cytoplasms from maize were never in parallel [11].

Based on this background, our intention was to determine whether the characteristics of CMS mitochondrial sequences of rice are comparable with those of maize or sugar beet. In rice, more than 60 types of CMS have been reported (see [20] or [21] for reviews); however, the mitochondrial sequence has not yet been revealed for any CMS rice. In this study, we employed two independent CMS lines, LD-CMS and CW-CMS, derived from the Burmese strain Lead rice (*Oryza sativa *L. ssp. *indica*) and from the Chinese wild rice strain W1 (*Oryza rufipogon *Griff.), respectively. Using the Nipponbare mitochondrial sequence as a reference, we chose the high-throughput pyrosequencing method to gather information about the rice CMS mitochondrial genomes. A number of genomic rearrangement events were detected between the two CMS mitochondria in comparison with that of Nipponbare.

## Results

### Sequence assemblies and assessment of genomic arrangements in CW-CMS and LD-CMS mitochondrial genomes

**Figure 1:**
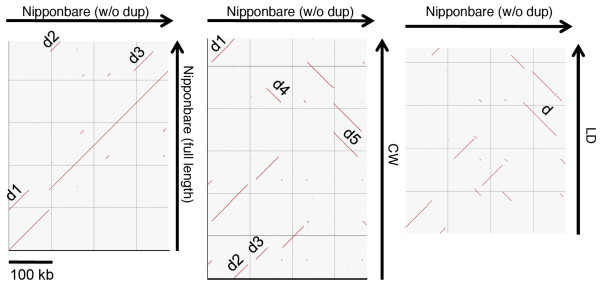
**Plots of syntenic regions of the rice mitochondrial genomes**. Nipponbare genome (without duplicates) on the X-axis, plotted against CW-CMS or LD-CMS genomes on the Y-axis. The letter 'd' accompanying the numbers indicates duplicated regions (>10,000 bp).

**Figure 2:**
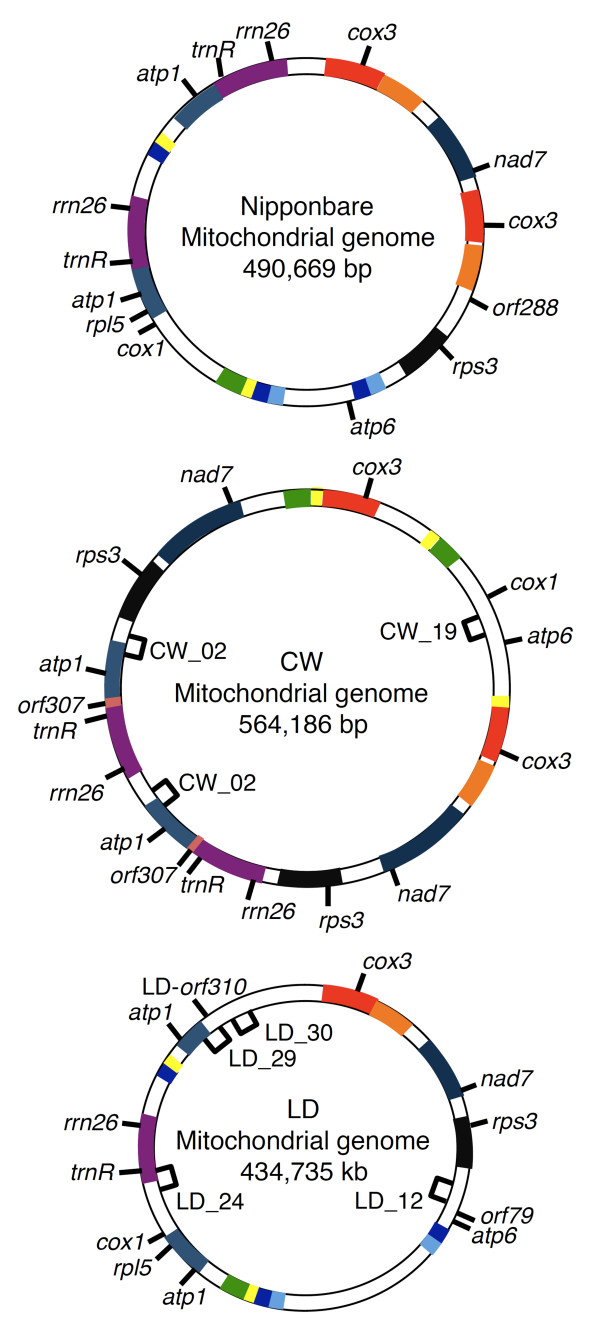
**Hypothetical master circles of the CW and LD mitochondrial genomes, and that of Nipponbare as a reference **[8]. Homologous regions are indicated by boxes of the same colors. Approximate regions amplified by the primers used in Additional file 6 are also indicated with brackets.

The CW-CMS mitochondrial genome was assembled to 559,045-bp single circular sequences, whereas the LD-CMS mitochondrial genome was predicted to have 434,735 bp when the master circle was hypothesized. When compared with the previously assembled Nipponbare (490,669 bp), PA64S (490,673 bp), and 93-11 (491,515 bp) [9] genomes, the sequence length of the two CMS lines differed greatly (Table 1). Thus, we speculated that the predicted mitochondrial genomic organization of the CMS lines is quite inconsistent with that of the known cultivars. We plotted the syntenic regions via the bl2seq algorithm using the Nipponbare sequence, excluding three large (>10,000 bp) duplicated regions (Figure 1). When the entire Nipponbare sequence including duplicated regions (Nipponbare full-length) was plotted against its truncated version (Nipponbare w/o dup.), we detected the presence of three large redundant regions in the same positions where we deleted duplications. However, when we plotted the sequences from the two CMS lines to the same reference genome (Nipponbare w/o dup.), we found that the syntenic regions were largely discrepant in their genomic positions (Figure 1). In comparison with Nipponbare, which has three large duplications (>10,000 bp), we predicted five duplications in the CW-CMS genome and one in the LD-CMS genome (Table 1, Figure 1). This feature is consistent with the observation in maize mitochondrial comparative sequence analysis that the large duplications accounted for most of the genome size increases in mitochondria [11], and the largest genome (CW-CMS: 559,045 bp) outnumbered the smallest genome (LD-CMS: 434,735 bp) in large duplication events. The number of recombination events was at least 15-fold higher in the CW-CMS genome compared to Nipponbare and at least 12-fold higher in the LD-CMS genome. The hypothetical master circle of CMS mitochondrial genomes is displayed in Figure 2.

**Table 1:** Summary of genomic organization.

	Nipponbare	PA64S	93-11	CW-CMS	LD-CMS
**Accession no**.	DQ167400	DQ167807	DQ167399	AP011076	AP011077
Origin of cytoplasm	*japonica*	*japonica*	*indica*	wild-rice	*indica*
Length	490,669	490,673	491,515	559,045	434,735
Synonymous mutations*	--	0	3***	1	0
Non-synonymous mutations*	--	0	2***	5	1
Large duplications (>10,000 bp)**	3	3	3	5	1
Recombination (vs Nipponbare)	--	0	0	15	12
Large InDels (>1,000 bp)	--	0	0	1	2

*In comparison with Nipponbare within genic regions.

** In comparison with Nipponbare (w/o dup.)

*** Referred in [9]

### Comparison of nucleotide sequences in genic regions

**Figure 3:**
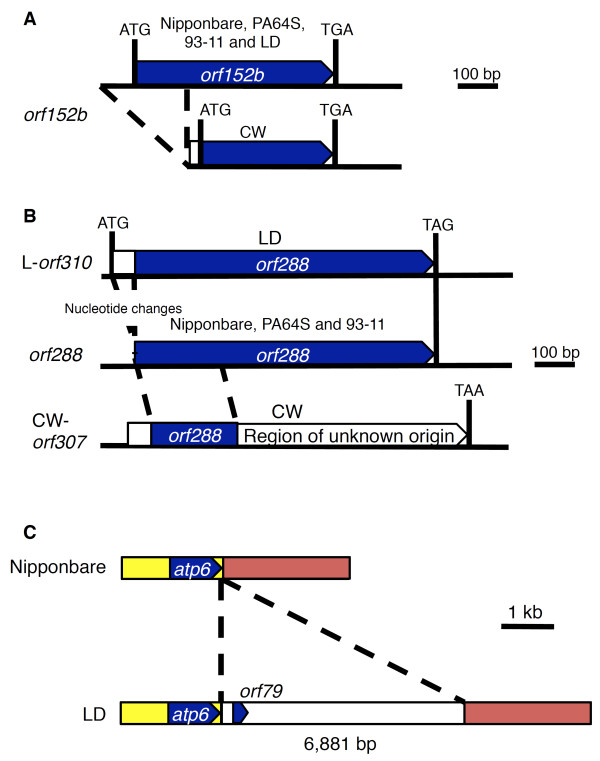
**Major changes in gene coding regions in the CMS lines**. **(A) **Structure of *orf152b*. The deletion of nucleotides in the N-terminal region shortened the ORF to a length of 92 amino acids in the CW-CMS genome. **(B) **Structures of *orf288*. Nucleotide extension at the N-terminal region seen in the LD-CMS genome caused the generation of a novel ORF predicted to encode a 310-amino-acid polypeptide (L-*orf310*). The chimeric structure of partial *orf288 *for N-terminal peptides and sequences of unknown origin generated the CW-*orf307 *gene in the CW-CMS genome. **(C) **The 6,881-bp alien sequence insertion in the downstream region of *atp6 *in the LD-CMS genome.

We compared nucleotide sequences among five mitochondrial genomes: Nipponbare, PA64S, 93-11, and the two CMS lines. First, 56 protein-encoding genes (including pseudo-genes), 22 tRNA genes (including pseudo-tRNA), and three rRNA genes found in Nipponbare were searched in the other genomes (list in Additional file 1, Table 2, Table 3). Including genes of unknown functions and ribosomal protein subunits, the appearances of the majority of the genes were constant among the five genomes. An exception was found for two ORFs which were missing from the CW-CMS genome. Due to the N-terminal deletion, the *orf152b *found in Nipponbare was shortened to an ORF of 92 amino acids in the CW-CMS genome (Table 3, Figure 3). Only the N-terminus 294 bp sequences of *orf288 *were present in CW-CMS (CW-*orf307*), whereas the *orf288*-like sequence was present but elongated to an ORF encoding 310 amino acids with a recombination event at the sequence corresponding to its N-terminal region in the LD-CMS genome (LD-*orf310*, Figure 3).

**Table 2:** Summary of SNPs in genic regions in comparison with Nipponbare.

	Numbers of synonymous mutations				Numbers of non-synonymous mutations			
	
Genes	CW	LD	PA64S	93-11	CW	LD	PA64S	93-11
*cox3*	--	--	--	2	--	--	--	1 (K3Q)*
*orf224*	1	--	--	1	1 (G3N)	--	--	1 (S186L)*
*rps1*	--	--	--	--	2** (R144G)	--	--	--
*rps2*	--	--	--	--	2 (K183N, F184Y)	--	--	--

* Two nucleotide mutations resulted in one amino acid exchange.

**Table 3:** Summary of InDels and other mutations in genic regions in comparison with Nipponbare.

	Numbers of deletions				Other mutations			
	
Genes	CW	LD	PA64S	93-11	CW	LD	PA64S	93-11
*cox3*	--	--	--	--	--	--	--	--
*orf152b*	--	--	--	--	N-terminal deletion	--	--	--
*orf176*	--	--	--	9	--	--	--	--
*orf288*	--	--	--	--	Partially present	orf310* (L162P)	--	--
*pseudo-rps14*	6	6	--	--	--	--	--	--

* ORF elongation by nucleotide changes in the N-terminal region. L162P is the amino acid change in *orf288 *basis.

We found four non-synonymous mutations in the CW-CMS genome (Table 2). Two point mutations caused a single amino acid change from arginine to glycine (R144G) in *rps1 *in the CW-CMS genome (Table 2). For *rps2*, two point mutations caused two amino acid changes, K183N and F184Y (Table 2). On the other hand, no amino acid changes were detected in the protein encoded by the LD-CMS genome in comparison to that of Nipponbare (Table 2), other than the 6-bp deletion in the pseudo-rps14 gene-like structure similarly observed in CW. The presence and the sequences of the electron chain subunit-encoding genes, *nad*, *cob*, *cox*, and *atp*, were completely conserved among the CMS lines and Nipponbare.

### Evaluation of known SNPs in 93-11 vs PA64S

As the genomic arrangements of the CMS lines and the previously sequenced cultivars were quite divergent (Figure 1), alignment of the whole genomes to detect SNPs or InDels was difficult. Thus, we decided to analyze 96 SNPs, 25 InDels, and three segmental sequence variations (SSV) detected by comparing 93-11 and PA64S in the study by Tian et al. [9]. The genotypes in the sequence variations are summarized in Additional files 2 and 3. Of the non-redundant 70 SNPs, only 59 SNPs were detectable in the CW-CMS and LD-CMS genomes, due to genomic deletions (Additional file 2). All of the SNP genotypes in the CW-CMS and LD-CMS genomes were the same (Additional file 2). Approximately 57% of the SNP genotypes were the same as those shared by the two *japonica *genomes (designated j); 14.3% were shared with the indica strain 93-11, 8.3% with PA64S (p), 2.9% with SNPs shared by 93-11 and Nipponbare (9/n), and 1.4% were unique to the two CMS genomes.

Non-redundant InDels and SSVs were also surveyed and, in parallel to the SNPs, the genotypes of the two CMS lines were completely identical (Additional file 3). Approximately 81% of the genotypes in the CMS lines were equal to *japonica *type, whereas 14.8% were equal to 93-11 type. One InDel was highly variable, and we were able to distinguish all lines in this region except for the CMS lines (InDel 200488, Additional file 3). From the above results, the CMS genomes were rather close to *japonica *genomes when only the nucleotide sequences were taken into consideration. Overall, the sequence variations that we could detect between the two CMS lines encompassed: the elongation of *orf288 *into *orf310 *in the LD-CMS genome, whereas *orf288 *was lost from the CW-CMS genome; the absence of *orf152b *from the CW-CMS genome; and mutations in *rps1 *and *rps2 *(Figure 3, Additional file 2). Direct sequence comparison of the syntenic regions of the CW-CMS and LD-CMS mitochondrial genomes revealed 43 SNPs and InDels in total. Comparison of the syntenic regions of the CW-CMS and Nipponbare genome revealed 206 SNPs and IndDels, although it is possible in this case that some of these differences may result from the different sequencing technologies employed. Thus the two CMS genomes were relatively similar to one another in comparison to the other mitochondrial genomes. The phylogenetic relationship estimated from all SNPs of both genic and non-genic regions is displayed as a dendrogram in Additional file 4.

### Gene structures unique in CMS lines

Similar to the studies that reported CMS-specific gene structures in the CMS mitochondrial genome of other species [3,11], we also found DNA rearrangements generating several CMS-specific structures. We previously identified a B-*atp6-orf79*-like structure in the LD-CMS mitochondrial genome, L-*atp6-orf79 *[22]. *orf79 *is the strongest MCAG candidate in BT-CMS rice [23-26]. Pyrosequencing enabled us to analyze the surrounding region of L-*atp6-orf79 *(Figure 3C). L-*atp6-orf79 *was on the long syntenic *atp6 *region with Nipponbare, but 6,881-bp alien sequences were inserted immediately after *atp6 *(Figure 3C). The insertion of 6,881 bp resulted in the generation of *orf79*. We have yet to determine the origin of the insertion sequences, but at least we were able to determine that the sequence was not present in Nipponbare, PA64S, 93-11, or the CW-CMS line. The L-*atp6-orf79 *transcripts are co-transcribed in the CMS line and the inter-genic region of the transcripts is cleaved by the action of the fertility restorer gene *Rf1 *[22]. The precise expression analysis of this L-*atp6-orf79 *locus was conducted in our previous study [22].

**Figure 4:**
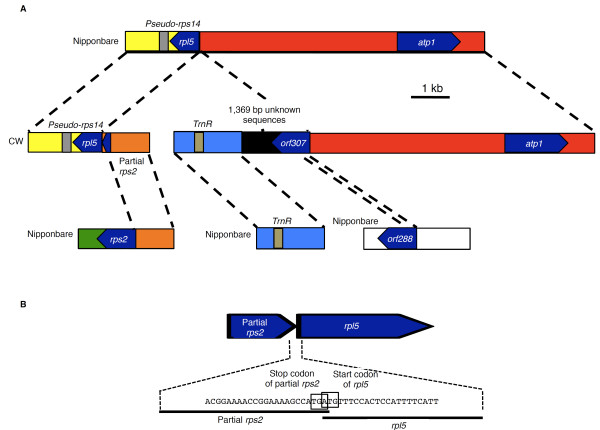
**Genomic structures around CW-*orf307 *and *rpl5***. **(A) **Large-scale genomic re-organization presumed to explain the evolution of the CW-*orf307 *region. Boxes of same colors indicate identical regions. **(B) **Schematic representation of the *rpl5 *locus in the CW-CMS mitochondrial genome. The A nucleotide in the TGA stop codon of partial *rps2 *inserted in the upstream region of *rpl5 *was equal to the A in the ATG start codon of *rpl5*. Thus, partial *rps2 *and *rpl5 *were not chimeric.

CW-CMS-specific genomic structures were found in the region around the *rpl5 *locus with exclusive genomic recombination involving the *orf288 *locus (Figure 4A). In Nipponbare, *rpl5 *was located upstream of *atp1*, 8.9 kb apart from it. In contrast, in the CW-CMS genome, the promoter region of *rpl5 *was substituted with other genomic regions by homologous recombination (Figure 4A). This recombination resulted in the insertion of nucleotides for 75 amino acids at the N-terminus of *rps2*, but the insertion was not considered to affect the *rpl5 *ORF structure because of the stop codon between partial *rps2 *and *rpl5 *(Figure 4B). Full-length *rps2 *was found in another location in the CW-CMS genome (data not shown).

**Figure 5:**
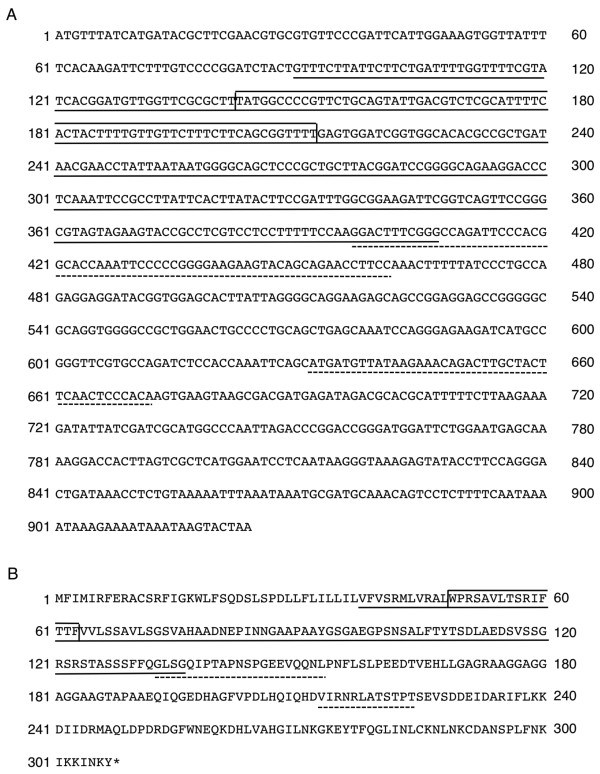
**Sequence of CW-*orf307***. **(A) **Nucleotide sequence of the predicted coding region. **(B) **Amino acid sequence of the predicted protein. The region identical to *orf288 *is underlined. The region identical to *cox2 *is boxed within the *orf288-*homologous region. Indicated by broken lines are the regions similar to *orf224*.

A rather interesting finding was the fate of the region around the *atp1 *locus because it was involved in the generation of the chimeric ORF gene, which was comprised of multiple recombination events (Figure 4A). The upstream region of *atp1 *was fused to a 294-bp sequence of *orf288 *corresponding to its N-terminal region (Figure 4A), and it was further followed by 1,369-bp unknown sequences and finally linked to the *trnR *region which is 26.0 kb away from *atp1 *in Nipponbare (Figure 4A). The 1,369-bp unknown sequence found in this locus was not found in the Nipponbare, PA64S, 93-11, or LD-CMS genomes, although some of its fragments showed 85% similarity to *orf224 *(Figure 5). As we stated previously, a part of *orf288 *was present in this region but the full-length copy was not present. The newly identified gene in this locus, possibly encoding a protein of 307 amino acids, was designated as CW-*orf307*. The structures of the *rpl5 *and CW-*orf307 *loci identified by pyrosequencing were verified by sequencing the lambda clones covering these regions (data not shown).

### Genomic structures unique in CMS lines

In order to evaluate the uniqueness of these genomic structures, distribution of the LD- and CW-specific structures and genes was surveyed in the National Institute of Agrobiological Sciences (NIAS, Japan) global rice core collection consisting of 69 accessions of worldwide cultivars [27]. This rice collection retains 90% of RFLP alleles detected in about 300 accessions selected based on the passport data from the whole rice collection (about 30,000 accessions) maintained at the NIAS Genebank [28]. Nine wild rice strains (*O. rufipogon*), which had previously been reported to carry a CMS cytoplasm [29], were also included in this study, so that 78 accessions were tested in total. Six primers able to amplify the contig linkage regions (described precisely in the Methods) unique in the two CMS lines in comparison with Nipponbare were used to detect each amplicon by PCR. Table 4 summarizes the results of PCR amplification and accessions were categorized into 12 mitochondrial haplogroups (A to L). No amplicon was observed in any of the accessions using primer pairs CW_02 or LD_24 (Table 4). Thus, the genomic rearrangement detected by primer pair CW_02, which corresponds to the region between the *atp1 *and *rrn26 *or *rps3 *genes in CW-CMS (Figure 2), and the rearrangement detected by the primer pair LD_24, which corresponds to the region between *cox1 *and *trnR *in LD-CMS (Figure 2), were completely unique to each CMS line. The range of the proportion of the accessions with amplification by four other primer pairs (CW_19, LD_12, LD_29, LD_30) was 3.8 - 21.8%. Similar to the genomic structure detected by primer pair CW_02, CW-*orf307 *was solely present in the CW-CMS line (Table 4). Excluding two *O. rufipogon *accessions (W1086 and W1092, Additional file 5), the presence of the *atp6-orf79 *structure was completely linked with the amplification pattern of LD_12, as expected from the closeness of the two regions (Table 4). Lastly, distribution of the *orf288-*like gene in rice accessions was assessed, as the gene was not present in the CW-CMS genome (Figure 3, Table 4). Thirty-five accessions carried the *orf288*-like structure, whereas no amplification was observed in the other lines.

**Table 4:** Summary of haplogroups distinguished by PCR amplification

	Contig linkage primer pairs*						Genes		
		
Haplogroups**	CW_02	CW_19	LD_12	LD_24	LD_29	LD_30	CW-*orf307*	*atp6-orf79*	*orf288*
A	-	-	-	-	-	-	-	-	+
B	-	-	-	-	-	-	-	-	-
C	-	+	-	-	-	-	-	-	-
D	-	-	+	-	+	+	-	+	-
E	-	-	-	-	+	+	-	-	-
F	-	+	-	-	-	-	-	-	+
G	-	-	-	-	-	+	-	-	+
H	-	-	+	-	-	+	-	+	-
I	-	-	-	-	+	+	-	-	+
J	-	-	+	-	+	-	-	+	-
K	-	+	-	-	+	+	-	-	+
L	-	-	-	-	+	+	-	+	-
CW	+	+	-	-	-	-	+	-	-
LD	-	-	+	+	+	+	-	+	+

*See Additional File 8 for details of the primers

**Full details of haplogroups are present in Additional file 5.

### Expression analysis of genes involved in the recombination events

We investigated whether the genes involved in the recombination to generate the chimeric CW-*orf307 *locus were affected in terms of their expression patterns. As a result, *cox1*, *orf284*, *orf165*, *atp1*, *cox2*, *trnR*, *rpl5*, and *rps2 *were expressed in the CW-CMS calli (Additional file 6), whereas the *orf224 *transcripts were extensively reduced in the CW-CMS line (Additional file 6). Due to the recombination event with *rps2*, *rpl5 *probably acquired a new promoter and, as a result, transcript elongation of *rpl5 *occurred (Figure 4B, Additional file 6).

**Figure 6:**
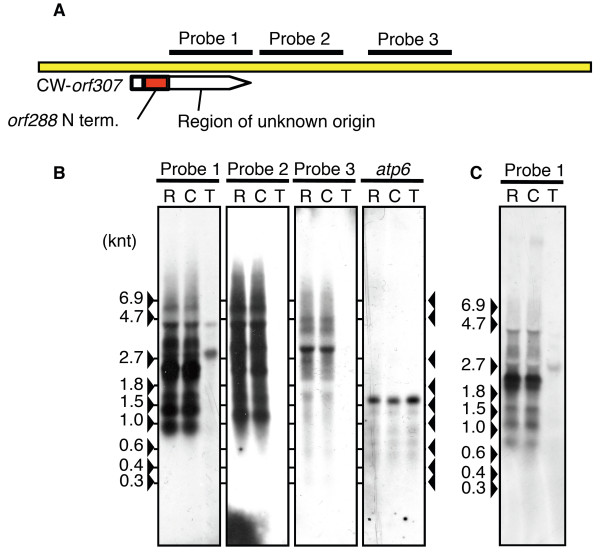
**Transcript detection around CW-*orf307 *locus by northern blot analysis**. **(A) **C-terminus of CW-*orf307 *and the downstream sequences were probed. **(B) **Northern blot analysis of mitochondrial RNA extracted from calli using probes 1 to 3. *atp6 *was used as the internal control. **(C) **Northern blot using probe 1 to detect CW-*orf307 *transcript using the total RNA extracted from anthers at the tricellular pollen stage. R, CWR (The restorer line carrying *Rf17 *and CW cytoplasm); C, CW-CMS line; T, Taichung 65.

For CW-*orf307*, the regions that do not include partial *orf288 *were used as probes (probes 1-3, Figure 6). We detected a faint band in Taichung 65, the *japonica *nuclear donor for the CW-CMS line, using probe 1. The detection of this slight signal in Taichung 65 might have occurred via the *orf224*-like region in probe 1 and the two regions showing more than 85% identity with *orf224 *(Figure 5). On the other hand, we detected a CW-mitochondria-specific RNA expression using probes 2 and 3, suggesting that the downstream regions of CW-*orf307 *were co-transcribed (Figure 6). Although we checked to see if the presence of the gametophytic restorer gene, *Rf17 *for CW-CMS, would have any effects on the transcript regulation of CW-*orf307 *in anthers, no differences in transcript patterns were observed between a CMS line and a CW-restorer line, CWR, carrying *Rf17 *and CW-cytoplasm (Figure 6C).

## Discussion

We obtained the entire mitochondrial genome sequences of two rice CMS lines of distinct origin. The master circle of the CW-CMS mitochondrial genome was predicted to contain 559,045 bp, whereas that of the LD-CMS genome had 434,735 bp. From a comparison with the recently assembled Nipponbare *(japonica*), PA64S (*japonica*), and 93-11 (*indica*) mitochondrial sequences [9], we found that the CMS genomes were exclusively rearranged (Figure 1). Based on the novel genomic rearrangements and unique ORFs discovered in this study, the mitochondrial haplogroups of the rice cultivars were divided into at least 12 groups (Table 4). It is highly likely that several mitochondrial genomic rearrangements occurred during rice breeding; apparently some rearrangements had deleted the CMS-specific genomic structures (Table 4). For instance, the strongest MCAG candidate of CW-CMS, CW-*orf307*, was absent from all of the other strains tested (Table 4, Additional file 5). The gene product of CW-*orf307 *was predicted to be strongly hydrophobic by SOSUI [30], which is consistent with the characteristics of previously known MCAG products [16-19]. Although we failed to detect a transcriptional alteration of CW-*orf307 *in the presence of *Rf17*, it is still possible that *Rf17 *could be related to translational regulation of CW-*orf307*. The presence of *orf79 *was observed in seven cultivars and in most of the *O. rufipogon *accessions that were previously reported to carry CMS cytoplasm [29], but *orf79 *was lost from the other cultivars presumably during rice breeding history (Table 4). The reason we speculate that *orf79 *was "lost" from majority of cultivars is because *O. rufipogon *genomes are generally considered as the ancestors of *O. sativa *cultivars.

The complete copies of *orf152b *and *orf288 *predicted in the Nipponbare genome were not present in the CW-CMS mitochondrial genome. Furthermore, a copy of *orf224 *was present in the CW-CMS genome, but its expression was unexpectedly suppressed (Additional file 6), possibly due to alteration of the transcriptional efficiency by the SNP 26 bp upstream of the initiation codon ATG or the two-nucleotide substitutions within the coding region (Table 2). As *orf152b *and *orf288 *were expressed in Nipponbare (S.F. and K.T. unpublished data), as was *orf224 *in this study (Additional file 6), we hypothesize that these ORFs are genes. Therefore, the genetic functions of *orf152b, orf288*, and *orf224 *were apparently missing or knocked down in the CW-CMS mitochondrial genome. Among these genes, the introduction of *orf288 *in the cultivars seemingly occurred during the rice breeding process. In fact, *orf288 *was present in only 35 accessions out of the 69 'core-collection' cultivars and in no accessions from *O. rufipogon*, indicating that the acquirement of *orf288 *at least had definitely occurred during the breeding history (Additional file 5). The absence of *orf288 *in approximately half of the cultivars indicates that this gene is obviously not necessary for pollen fertility. Thus the possibility that a loss of function of *orf288 *in the CW-CMS line is causing CMS is not likely, because again absence of *orf288 *apparently does not induce male sterility in such cultivars lacking *orf288*. Whether *orf288 *itself can be directly involved in CMS is still an open question, but it may be postulated that the presence of *orf288 *has contributed to the evolution of a new chimeric CMS gene as a segment of approximately 70 amino acids of *orf288 *is identical to *cox2 *(data not shown), persuading us that *orf288 *is a chimeric gene. Therefore, we conclude that compelling evidence of the 'death' of MCAG via genomic rearrangements during cultivation was demonstrated in this study and, contrarily, that the generation of genes such as *orf288 *may be part of the 'birth' process. The 'death' idea is also supported by the fact that the *atp6-orf79-*like structure was lost from a large proportion of the cultivars (Additional file 5).

The actual mechanism underlying the process of MCAG is considered to be substoichiometric shifting (SSS), which involves the idea that the copy numbers of multipartite mitochondrial genomic molecules (subgenomes) change over generations [31,32]. Interactive recombinations between repeat regions within subgenomes create novel genomic rearrangements, and these novel subgenomes may be present in low copy numbers at first, but they can proliferate and become the majority in subsequent generations by SSS [33]. It has actually been proven that the knockdown of the homologue of the *Escherichia coli *mismatch repair component in tomato and tobacco unsuppressed the mitochondrial recombination events, and that it eventually caused artificial CMS possibly intermediated by SSS [34]. Therefore, quantitative measurement of the genomic rearrangements we found in the above experiments should provide us with a clearer picture of how the evolution of MCAG took place during rice breeding.

A CMS genome determination study of sugar beet reported a few CMS-specific chimeric structures, other than the best MCAG candidate, *preSatp6 *[3,18]. Maize CMS sequencing detected one chimeric ORF other than MCAG T-*urf13 *in CMS-T; none were detected other than the best MCAG candidate *orf77 *in CMS-S; and none were detected in CMS-C under the 70-amino-acid cut-off criterion [11]. Whether this is meaningful or not, we found 480 ORFs in the Nipponbare genome, 591 in the CW-CMS genome, and 456 in the LD-CMS genome, using the same 70-amino-acid cut-off criterion. Out of the ones that were not present in Nipponbare, none except for the ones we listed (CW-*orf307 *and LD-*orf79*) contained a significant portion of the other known genes. Whether any one of these kinds of ORFs is actually related to CMS is still unknown, and it should be emphasized again that Nipponbare as well contains genes that were not described as related to known mitochondrial biological processes but are actually transcribed. The transcribed proportion of these predicted ORFs is undetermined but, based on our current knowledge, detection of any protein products of these genes has not yet been reported [35,36].

On the other hand, the generation and maintenance of a novel chimeric ORF may be allowed by the presence of certain nuclear genes. Possible candidates of such nuclear-encoded genes are pentatricopeptide repeat (PPR)-motif containing genes as discussed below. PPRs are now undoubtedly considered to be involved in post-transcriptional gene regulation events, mostly in plant organelles [37-40]. In higher plant species 400 to 600 PPR genes are encoded, and there is concrete evidence demonstrating that orthologous PPR pairs exist between rice and Arabidopsis [39]. Most of the *Rf *genes (nuclear-encoded genes responsible for fertility restoration of CMS) identified to date have been demonstrated to encode a PPR protein [25,41-48]. PPRs encoded by *Rf *genes (*Rf-PPR*) are exceptional because they are not conserved among the species [39]; they are curiously present as heavily duplicated clusters of *Rf-PPR-*like genes in the genome. This feature resembles that of diversifying selection acting on the evolution of the disease resistance *R *genes [49]. There have been a few implications that *Rf-PPR*s are actually positively selected [50,51]. One perspective involves the expectation that some *Rf-PPR*-like genes may be responsible for preventing protein accumulations from certain predicted ORFs that are transcribed but untranslated [40]. There has been no concrete evidence demonstrating that the evolution of *Rf-PPR*s is related to the generation of novel ORFs in mitochondria; however, there certainly must be a selective constraint to the diversifying selection of the *Rf-PPR*s, in the same manner as there are pathogens restricting the evolution of the *R *genes. Our current study suggested that rice mitochondrial genomic rearrangement is an on-going event, and it seems quite reasonable to consider that frequent novel ORF generation as a consequence is the driving force of *Rf-PPR *diversification.

## Conclusion

We have shown that the mitochondrial genomes of the two CMS lines are greatly reorganized in comparison with that of Nipponbare. There appear to be a few genomic structures that are relevant to CMS, and finding these structures within rice cultivars and wild accessions provided us with the idea that genomic recombination events during rice breeding history diminished these unique genomic structures of CMS and, on the other hand, presumably gave rise to novel ORFs.

## Methods

### Plant materials

The origins of two independent rice CMS lines, LD-CMS and CW-CMS, were described in our previous studies [22,52]. LD-CMS is derived from the Burmese cultivar Lead rice (*Oryza sativa *L. ssp. *indica*) [53], and CW-CMS originates from the Chinese wild rice strain W1 (*Oryza rufipogon *Griff.) [54]. The nuclear donor maintainer line Taichung 65 (*japonica*) was used as the control for the RNA expression experiment. The development of a restorer line for CW-CMS, CWR, carrying the fertility restorer gene *Rf17 *which functions in a gametophytic manner [52], was described in our previous studies [52,55]. The NIAS global rice core collection [27] was obtained from the National Institute of Agrobiological Sciences Genebank (Tsukuba, Japan). *O. rufipogon *accessions were obtained from the National Institute of Genetics (Mishima, Japan).

### Mitochondrial DNA extraction

Mitochondria were purified on sucrose gradients, as described by Tanaka et al. [56]. The DNeasy plant mini kit (QIAGEN, Hilden, Germany) was used to extract DNA from the mitochondrial fraction.

### Sequence assembly

Pyrosequencing was conducted on the GS-FLX system (Roche, Basel, Switzerland) in TaKaRa-Bio (Otsu, Japan). We obtained 15,965,332-bp nucleotides for the CW-CMS genome and 15,020,464-bp nucleotides for the LD-CMS genome. Sequences were assembled to the average of 5,123-bp-long 824 contigs for the CW-CMS genome, and 4,966-bp-long 1,188 contigs for the LD-CMS genome, using the GS De Novo Assembler Software (Roche). The average sequence depth, which was defined as the sum of redundancies of each base divided by the length of the respective contig, was 41.74 for the CW-CMS genome and 44.75 for the LD-CMS genome. As many turning points and false linkages were considered, these contigs were manually assembled by the following criteria. (1) The depth of the bridging contigs had to be over 15, as bridging contigs with a depth under 15 often linked 'dead-end' contigs that were only linked to one another. Also, bridging contigs with a depth under 15 frequently linked fragments that were considered to be nuclear contaminants. (2) 'Dead-end' contigs were ignored. In addition, depth values of these 'dead-end' contigs (<15) were lower than those of non-dead-end contigs (>15). (3) Contigs that matched 100% with the Nipponbare or 93-11 nuclear genomic sequences were ignored as they were considered to be nuclear contaminants and, as in the case of 'dead-end' contigs, depth values of these contigs were always under 15. (4) Plasmid-like sequences, corresponding to B1, B2, B3, and B4 reported to be present in rice mitochondria [57,58], were deposited to DDBJ (under accession nos. AB523794 to AB523802), but they were not analyzed in this study. Their characteristics will be presented elsewhere in detail.

These criteria left us with 19 contigs with an average length of 17,877 bp for the CW-CMS genome, and 24 contigs with an average length of 14,828 bp for the LD-CMS genome. Following these steps, the candidate linkage of the contigs was validated by PCR analysis (Additional file 7, and see Additional file 8 for the primer sets). Based on the linkage information of the contigs confirmed as well by PCR analysis (depicted in Additional files 9 and 10), master circles were developed for each genome by a 'parsimonious' method, so that each contig appeared at least once to construct the smallest genome. For the CW mitochondrial genome, there were eight alternative master circle patterns, and 64 patterns for the LD genome. In this paper, one pattern of the master circle was chosen for brevity of presentation (Additional file 11).

The BLASTN program in NCBI tools [59] was used to assign these contigs to the reference Nipponbare mitochondrial genome (DQ167400; described in [9]), and BLASTN was also used to eliminate nuclear genomic contaminations for the above criteria. Handling of the sequences was performed by Sequencher v4.2.2 software (Gene Codes Corporation, Ann Harbor, Minnesota). The Genomematcher v1.2 program [60] was used to plot the sequenced genomes with the reference Nipponbare genome by bl2seq. To avoid the possibility of detecting duplicated regions more than twice, the Nipponbare genome without large duplicated regions (>10,000 bp) synthesized for this study was referenced. Annotated sequences were deposited to DDBJ under accession nos. [DDBJ:AP011076] for CW-CMS and [DDBJ:AP011077] for LD-CMS.

### Sequence comparison

Known mitochondrial genes in Nipponbare were BLASTN searched in the CW-CMS and LD-CMS genomes, and SNPs and Indels were detected. ORFs were attached to the newly sequenced genomes by the est2 genome program included in the EMBOSS v6.0.0 package [61]. Fifty-six protein-encoding genes (including pseudo-genes), 22 tRNA genes (including pseudo-tRNA) and three rRNA genes analyzed in this study are listed in Additional file 1. To obtain information for the genes that are not present in the Nipponbare genome, gene predictions were performed using the ORF Finder program [59] and Genemark.hmm for the Prokaryotes v2.4 program [62]. Six-frame-translation was performed using the getorf software included in the EMBOSS v6.0.0 package [61]. Since MCAG found in rice BT-CMS mitochondria genome encoded a protein of 79 amino acids, the ORFs predicted to encode peptides shorter than 70 amino acids were ignored.

Information on SNPs and InDels among Nipponbare, PA64S (DQ167807), and 93-11 (DQ167399) was obtained from the study by Tian et al. [9]. We considered it difficult to directly compare the pyrosequence data obtained in this study to that of the sequences obtained by shotgun sequencing in Tian et al. [9] because differences in sequence accuracies between the two strategies might be significant. Thus, instead, known SNPs and InDels in 93-11 vs PA64S [9] were surveyed in LD-CMS and CW-CMS genomes. Phylogenetic analyses of the mitochondrial genomes were performed using RAxML v 7.04 [63].

### Construction and screening of the lambda library

The lambda library was constructed from total DNA isolated from the leaves of CWR, carrying CW cytoplasm, by the CTAB method [64]. DNA partially digested by *Sau3A*I (TaKaRa-Bio) was ligated into λGEM-11 *Xho*I half-site arms (Promega, Madison, Wisconsin) using Ligation high (TOYOBO, Osaka, Japan), and packaged using Gigapack III Gold packaging extract (Stratagene, La Jolla, California) according to the manufacturer's protocol. Lambda clones were screened using the probes synthesized from PCR and primer pairs 5'-AGTACCAAAAGCTGCCTCTG-3' and 5'-TTTCCCCCTCATCTTTTAGC-3' for the *rpl5 *region, and 5'-GCATGATGATCCGATAATC-3' and 5'-TAGGTTCGTTATCAGCGG-3' for the CW-*orf307 *region, respectively. Inserts in isolated clones were subcloned into pBluescript SK- after digestion by *Bam*HI, *Sac*I, *Sal*I, or *Xba*I (Takara-Bio, Otsu, Japan), and sequenced by the CEQ 8000 Genetic analysis system (Beckman Coulter, Fullerton, California). As a result of sequencing 20,513 bp, two mismatches were detected in comparison with pyrosequencing, thus the accuracy of pyrosequencing was estimated as 99.99%.

### Expression analysis

Mitochondrial RNA was extracted from calli using the typical phenol/chloroform RNA isolation method. For anthers at the tricellular pollen stage, total RNA was extracted using the same method. Mitochondrial RNA (2.5 μg) was electrophoresed on 1% agarose gel after denaturation by formamide treatment and transferred to a nylon membrane. Primers used to design probes for northern hybridization are listed in Additional file 1.

## List of abbreviations

CMS: cytoplasmic male sterility; InDel: insertion/deletion; MCAG: Mitochondrial cytoplasmic male sterility associated gene; PPR: pentatricopeptide repeat; Rf: restorer of fertility; SNP: single nucleotide polymorphism; SSV: segmental sequence variation.

## Authors' contributions

SF designed the study, carried out the experiments, performed sequence analysis, and drafted the manuscript. TK and MY participated in the experiments. KT conceived and supervised the work and edited the manuscript. All authors read and approved the final manuscript.

## Supplementary Material

Additional file 1 **Lists of genes and the corresponding primers used for the northern blot analysis**. Lists of mitochondrial genes in Nipponbare and the primers used for the northern blot analysis in Figure 6 and Additional file 6. Click here for file

Additional file 2 **Comparison of SNP genotypes among *japonica *mitochondrial genomes Nipponbare, PA64S, and indica 93-11**. Nucleotide positions in 93-11 are displayed. Genotypes in CMS are: j, *japonica *type; 9, 93-11 type; n, Nipponbare type; p, PA64S type; cms, CMS specific; -, no syntenic region present in the CMS lines. SNPs in the genic regions are given in the last column. Click here for file

Additional file 3 **Comparison of InDel and segmental sequence variation (SSV) genotypes among *japonica *mitochondrial genomes Nipponbare, PA64S, and indica 93-11**. Nucleotide positions in 93-11 are displayed. Genotypes are: j, *japonica *type; 9, 93-11 type; n, Nipponbare type; p, PA64S type; c, CMS specific. Sequence variations in the genic regions are given in the last column. *Nipponbare vs 93-11 InDels detected in the genomic study by Tian et al. (2006) [9]. Nucleotide positions in 93-11 are indicated. **Only CW-CMS and LD-CMS genomes retained the same sequences in this region, and other genomes possessed different sequences. Click here for file

Additional file 4 **Phylogenetic relationship of the five rice mitochondrial genomes**. All of the sequence variations were used to generate the maximum-likelihood inference-based dendrogram. Bar indicates the rate of nucleotide substitution per site. Click here for file

Additional file 5 **PCR amplification of the CMS unique genomic structure and genes**. PCR amplification positive (+) and negative (-) in each rice core-collection accession for each primer pair. * Refer to Figure 2, Additional files 8 and 11 for information on the primers. ** Refer to [28] for details. Click here for file

Additional file 6 **Transcript detection of genes involved in the evolution of the CW-*orf307 *locus**. Transcripts were detected by northern blot analysis using mitochondrial RNA extracted from calli. Genes involved in the *rpl5 *region (*orf165, orf284, cox1, pseudo-rps14, rpl5*, and *rps2*) and genes involved in CW-*orf307 *(*atp1, orf288, orf224, cox2*, and *trnR*). C-terminal region of *rps2 *was probed to avoid detecting partial *rps2 *present in the *rpl5 *locus. Also, C-terminal *orf288, orf224*, and *cox2 *were probed to avoid detecting the fragments in the *CW-orf307 *transcript. The transcript abundance of *atp6 *was used as the internal control, and the *atp6 *transcript level reflected the ribosomal RNA band intensities detected by ethidium bromide staining. R, CWR (The restorer line carrying *Rf17 *and CW cytoplasm); C, CW-CMS line; T, Taichung 65. Click here for file

Additional file 7 **Validation of contig linkage by PCR analysis**. Lane numbers refer to primer pairs in Additional file 8. Click here for file

Additional file 8 **Information on the primers used for the PCR analysis to validate the linkage between the contigs**. Information on the primers used for the PCR analysis in Additional file 7. Click here for file

Additional file 9 **Possible linkage of the contigs validated by PCR analysis for the CW-CMS mitochondrial genome**. Numbers within the arrows indicate the contig accession numbers, whereas numbers above indicate the sequence depth of each contig. The yellow color indicates the contigs that appear twice in our master circle, and the blue color indicates the contigs that appear three times. Capital letters represent contig linkages. Click here for file

Additional file 10 **Possible linkages of the contigs validated by PCR analysis for the LD-CMS mitochondrial genome**. See the legend for Additional file 9 for descriptions. Click here for file

Additional file 11 **Order and position of the contigs aligned in the master circles**. Sequences in this order were deposited to DDBJ (CW-CMS, AP011076; LD-CMS, AP011077). Click here for file
