# Supplementary figures and images for: Discovery of global genomic re-organization based on comparison of two newly sequenced rice mitochondrial genomes with cytoplasmic male sterility-related genes
Source: BMC Genomics. 2010 Mar 29;11:209. doi: 10.1186/1471-2164-11-209 (PMC2851602; doi:10.1186/1471-2164-11-209)

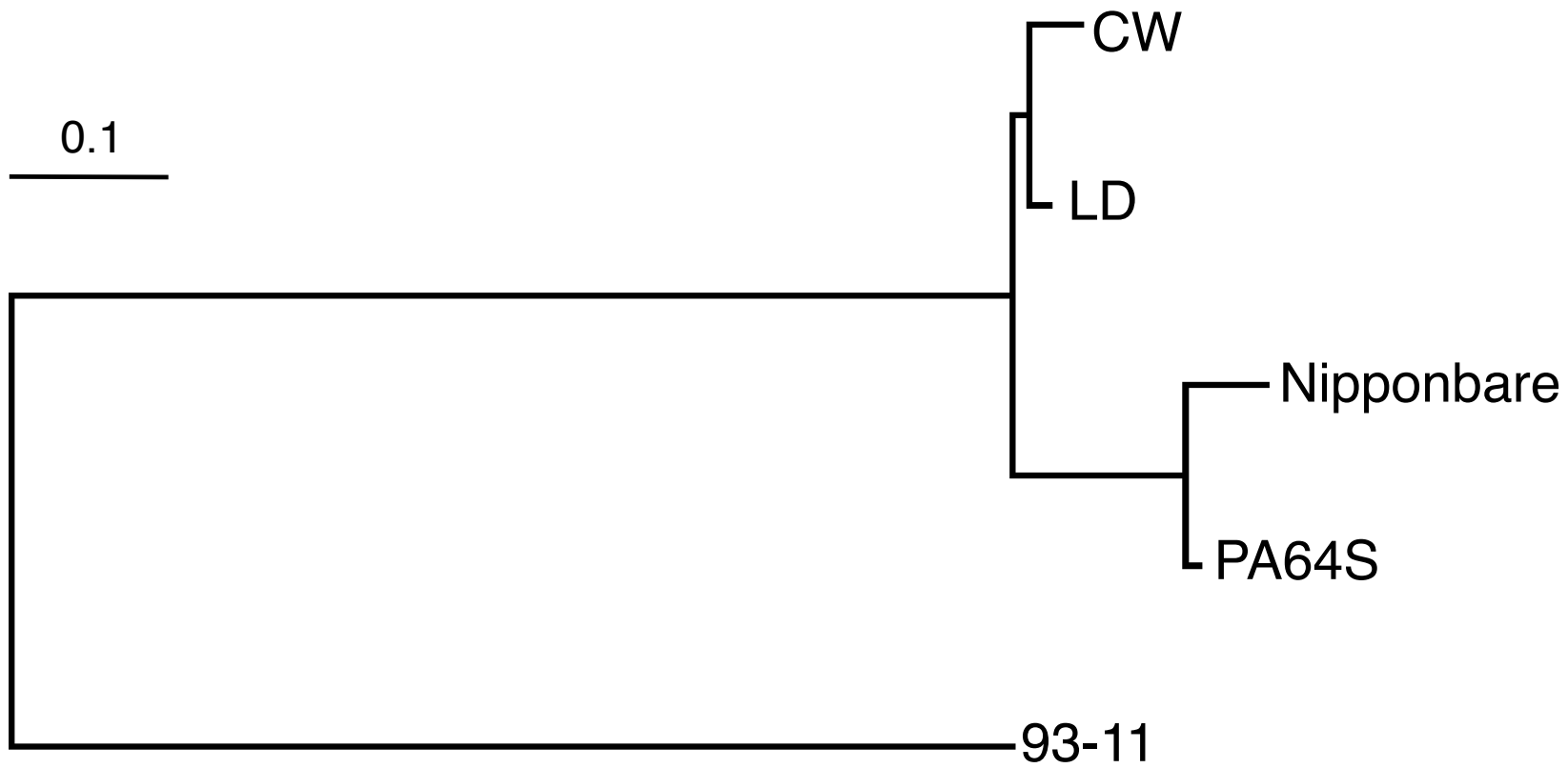

Supplement: Additional file 4 — Phylogenetic relationship of the five rice mitochondrial genomes. All of the sequence variations were used to generate the maximum-likelihood inference-based dendrogram. Bar indicates the rate of nucleotide substitution per site. [file 1471-2164-11-209-S4.PDF]

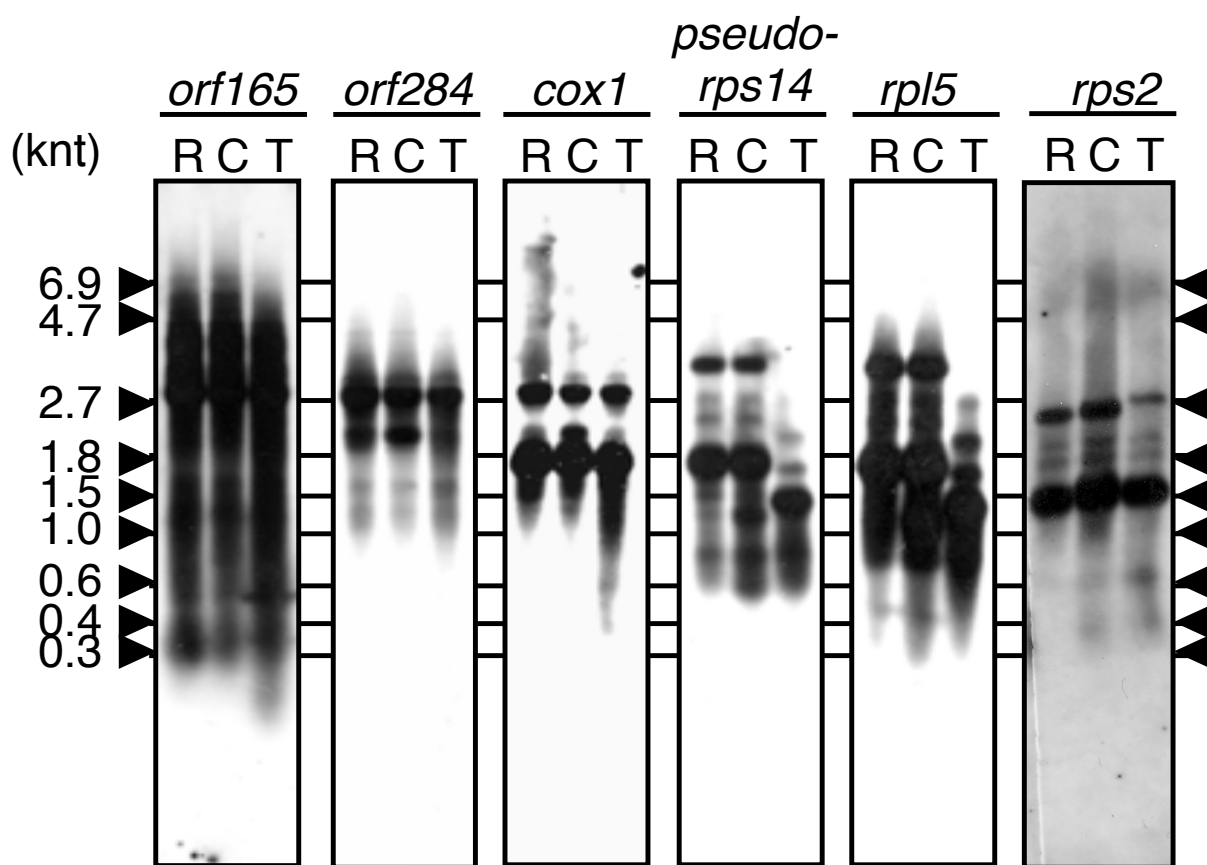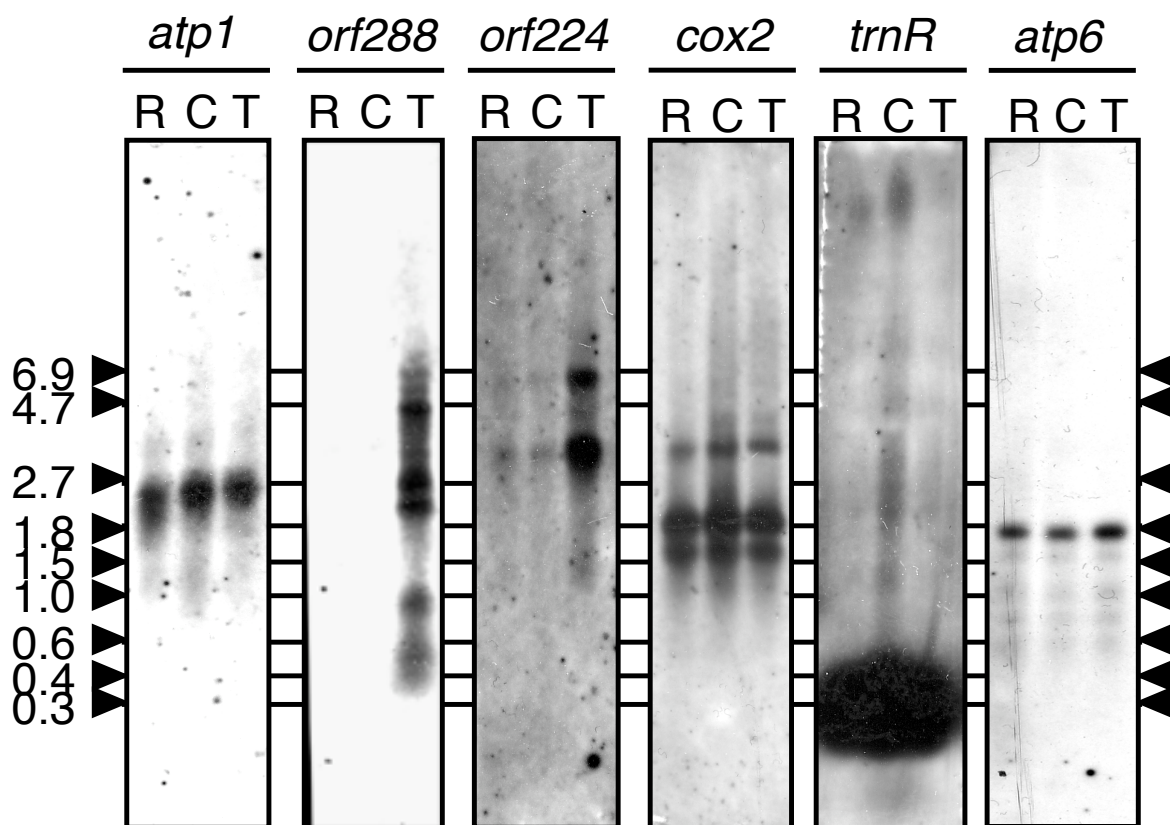

Supplement: Additional file 6 — Transcript detection of genes involved in the evolution of the CW-orf307 locus. Transcripts were detected by northern blot analysis using mitochondrial RNA extracted from calli. Genes involved in the rpl5 region (orf165, orf284, cox1, pseudo-rps14, rpl5, and rps2) and genes involved in CW-orf307 (atp1, orf288, orf224, cox2, and trnR). C-terminal region of rps2 was probed to avoid detecting partial rps2 present in the rpl5 locus. Also, C-terminal orf288, orf224, and cox2 were probed to avoid detecting the fragments in the CW-orf307 transcript. The transcript abundance of atp6 was used as the internal control, and the atp6 transcript level reflected the ribosomal RNA band intensities detected by ethidium bromide staining. R, CWR (The restorer line carrying Rf17 and CW cytoplasm); C, CW-CMS line; T, Taichung 65. [file 1471-2164-11-209-S6.PDF]

CW-CMS

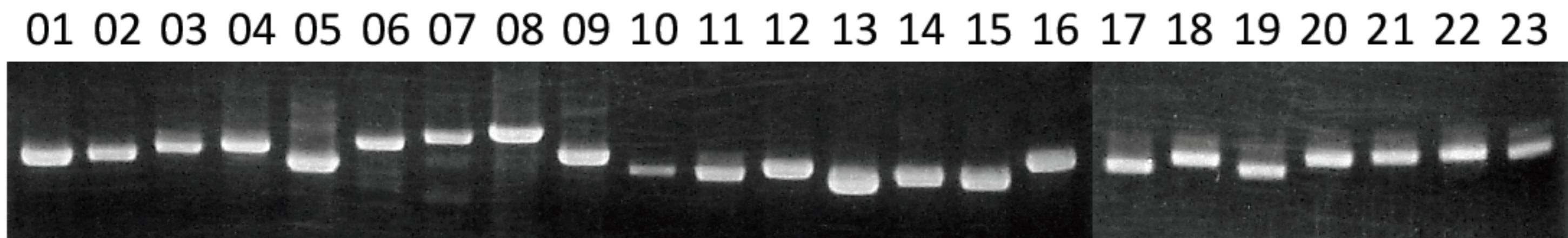

LD-CMS

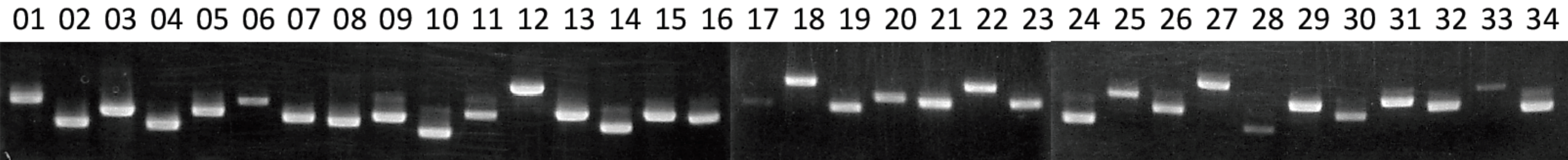

Supplement: Additional file 7 — Validation of contig linkage by PCR analysis. Lane numbers refer to primer pairs in Additional file 8. [file 1471-2164-11-209-S7.PDF]

# CW-CMS

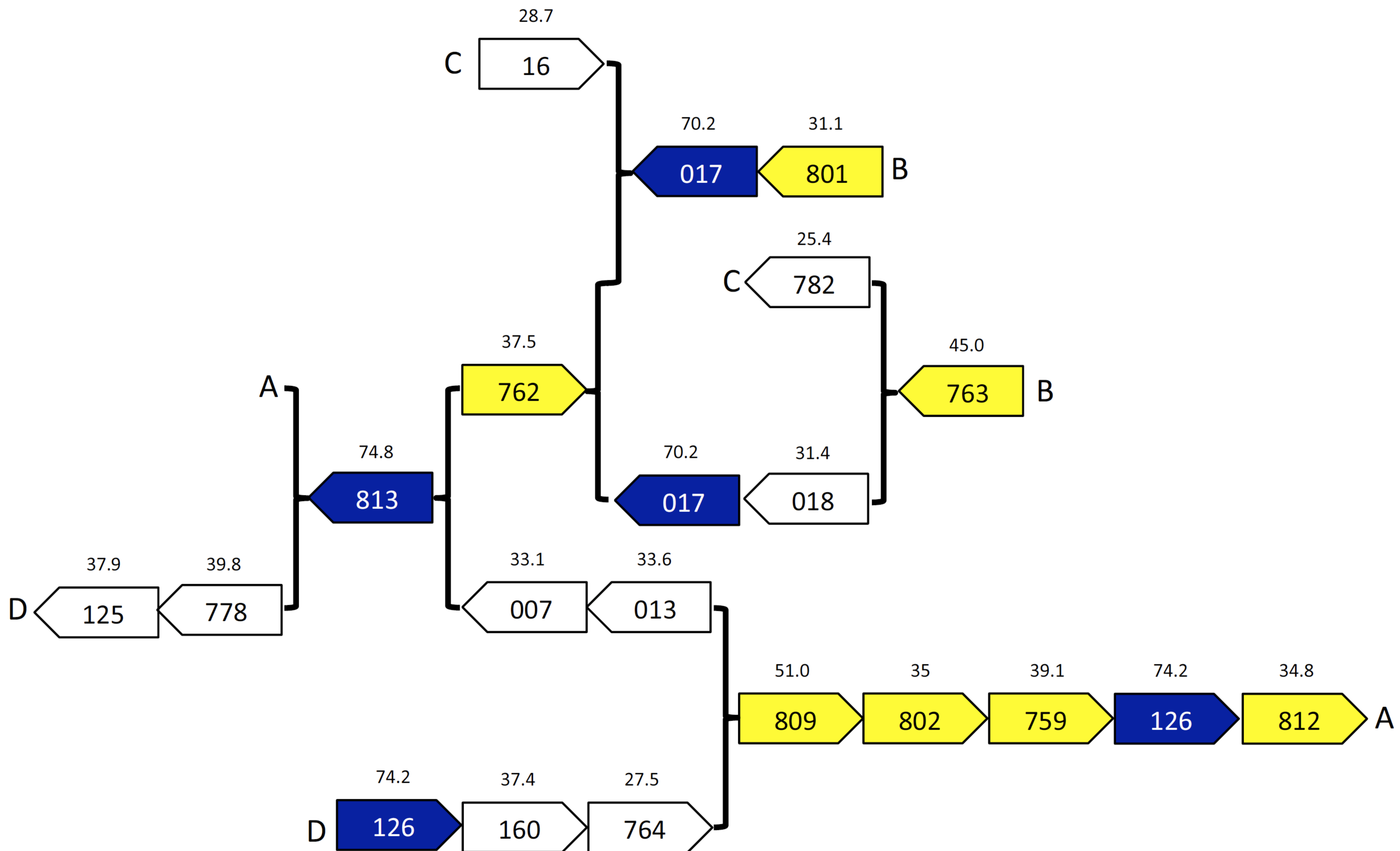

Supplement: Additional file 9 — Possible linkage of the contigs validated by PCR analysis for the CW-CMS mitochondrial genome. Numbers within the arrows indicate the contig accession numbers, whereas numbers above indicate the sequence depth of each contig. The yellow color indicates the contigs that appear twice in our master circle, and the blue color indicates the contigs that appear three times. Capital letters represent contig linkages. [file 1471-2164-11-209-S9.PDF]

LD-CMS

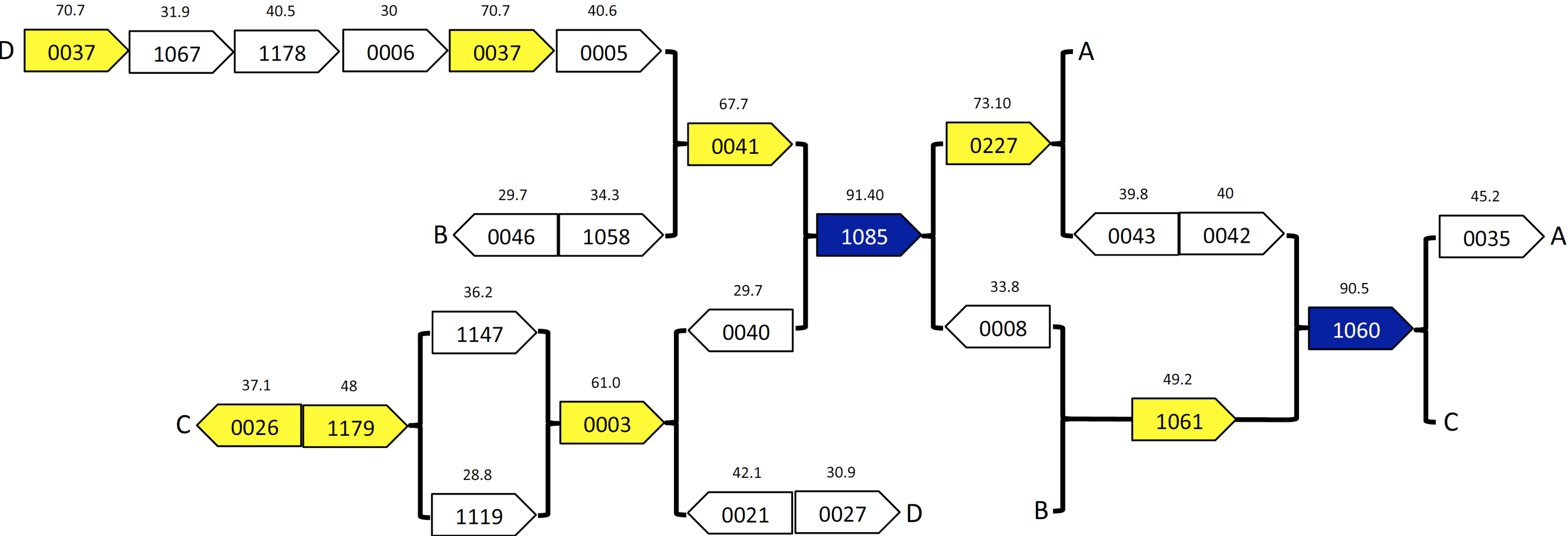

Supplement: Additional file 10 — Possible linkages of the contigs validated by PCR analysis for the LD-CMS mitochondrial genome. See the legend for Additional file 9 for descriptions. [file 1471-2164-11-209-S10.PDF]
